# Efficacy of the vegetative cells of *Lysinibacillus sphaericus* for biological control of insecticide-resistant *Aedes aegypti*

**DOI:** 10.1186/s13071-017-2171-z

**Published:** 2017-05-10

**Authors:** Paula Andrea Rojas-Pinzón, Jenny Dussán

**Affiliations:** Departamento de Ciencias Biológicas, Centro de Investigaciones Microbiológicas (CIMIC), Universidad de los Andes, Carrera 1 No. 18 A – 10, J-206, Bogotá, Colombia

**Keywords:** *Lysinibacillus sphaericus*, Vegetative cell, *Aedes aegypti*, Field-collected strain, Biological control

## Abstract

**Background:**

The control of *Aedes aegypti* is usually based on chemical insecticides, but the overuse of these compounds has led to increased resistance. The binary toxin produced by *Lysinibacillus sphaericus* in the final stages of sporulation is used for mosquito control due to its specificity against the culicid larvae; however, it has been proved that *Ae. aegypti* is refractory for this toxin. Currently, there is no evidence of the use of *L. sphaericus* vegetative cells for mosquito biocontrol. Therefore, in this study, the vegetative cells of three *L. sphaericus* strains were assessed against a field-collected *Ae. aegypti,* resistant to temephos, and the reference Rockefeller strain.

**Results:**

Vegetative cells of *L. sphaericus* 2362, III(3)7 and OT4b.25 produced between 90% and 100% of larvae mortality in the reference Rockefeller strain. Effective concentrations of each *L. sphaericus* strain for the four larval stages ranged from 1.4 to 2 × 10^7^ CFU/ml. Likewise, a consortium of *L. sphaericus* assessed against a field-collected *Ae. aegypti* resistant to temephos and the Rockefeller strain caused 90% of larvae mortality. Concentrations of *L. sphaericus* consortium that resulted in larvae mortality of field-collected and Rockefeller *Ae. aegypti* ranged from 1.7 to 2.5 × 10^7^ CFU/ml. The vegetative cells of *L. sphaericus* have no effect on the *Ae. aegypti* eggs and pupae.

**Conclusions:**

The vegetative cells of *L. sphaericus* are effective against *Ae. aegypti* larvae, meaning that it could be used in the biological control of these mosquito species. Since the *L. sphaericus* consortium was effective against temephos-resistant *Ae. aegypti*, vegetative cells could be an alternative to overcome insecticide-resistant populations. Further studies, should be conducted to reveal the mode of action and the toxic principle of *L. sphaericus* vegetative cells.

## Background

*Aedes aegypti* is the vector of major tropical diseases such as dengue, chikungunya, and Zika. The control of *Ae. aegypti* populations is mostly based on the application of chemical insecticides. However, the overuse of compounds such as DDT, malathion, and temephos has led to the development of insecticide resistance [1, 2]. Another issue to be considered is the negative ecological effect of these insecticides [3]. Hence, biological control can be a more specific and safer alternative in maintaining low levels of mosquito populations, and as such reducing the incidence of vector-borne diseases [4].

Colombia is a propitious harbour of major vectors such as *Culex* spp., *Anopheles* spp. and *Aedes* spp., and thus, an endemic country for the diseases transmitted by these mosquitoes [2]. Since the re-invasion of *Ae. aegypti* in the 1960s, prevention against the diseases transmitted by this mosquito is based on the use of chemical insecticides [5]. Although biological control of *Ae. aegypti* has not been implemented in Colombia, several *Lysinibacillus sphaericus* strains, isolated from various regions, exhibit a promising larvicidal activity to be used in mosquito control [6]. The toxic activity of some native strains was assessed previously against *C. quinquefasciatus* by Lozano and Dussán [7]. Vegetative cells of *L. sphaericus* strains OT4b.25, III(3)7, and the World Health Organization reference strain 2362 caused over 70% mortality against third-instar larvae of *C. quinquefasciatus* and presented metal tolerance suggesting a potential use of *L. sphaericus* in the biological control of mosquitoes in polluted environments [7].

*Lysinibacillus sphaericus* is a bacterium with entomopathogenic activity, attributed mainly to the binary (Bin) toxin, which is produced in the final stages of sporulation [8]. After ingestion of crystals containing the two polypeptides comprising the binary toxin, these are solubilized and activated in the larval midgut where cytopathological effects take place [9, 10]. The effects of *L. sphaericus* on mosquito larvae are well known given the specificity of the Bin toxin on *Culex* spp. and *Anopheles* spp. midgut receptors [11]. Although *Ae. aegypti* is closely related to these culicids, it is refractory to the *L. sphaericus* spore toxins [12, 13].

Other less toxic *L. sphaericus* proteins are the Mtx 1, 2 and 3 toxins, and the S-layer protein. The Mtx toxins are produced in the vegetative cells but are degraded by proteases at the late log phase [14]. Separately, the S-layer protein is a paracrystalline array that covers the entire surface and can be located on the vegetative cell or inside the spore [15]. In addition to its function in shape maintenance of vegetative cells, this purified protein exhibit entomopathogenic activity against *Culex quinquefasciatus* and *Ae. aegypti* larvae [16, 17]. Recombinant Mtx toxins and S-layer protein have been assessed against *Ae. aegypti* larvae [16, 18], but there are no reports regarding the whole vegetative cell. Recent genomic studies have revealed that some *L. sphaericus* strains have hemolysins and chitinases genes, which could play an important role in the entomopathogenic activity of these strains [19–21].

Resistance to commonly used insecticides such as temephos, malathion and deltamethrin have been reported in Brazil [22], Cuba [23] and Colombia [24]. Given that the emergence of resistant mosquito populations is a major concern in public health, several studies have been conducted to assess the current susceptibility of mosquito populations to chemical insecticides. In Colombia, reports indicate resistance in some areas such as Cundinamarca, where a field population of *Ae. aegypti* showed 1.2% mortality at 24 h, and consequently, it was determined to be temephos-resistant [25].

Several studies have shown the toxic activity of *L. sphaericus* crystal-spores against some mosquito vectors, but vegetative cells action has not been demonstrated. In this study, vegetative cells of *L. sphaericus* OT4b.25, III(3)7, and 2362 (WHO reference) strains were assessed against a temephos-resistant, the field-collected strain of *Ae. aegypti* and the Rockefeller reference strain.

## Methods

### *Lysinibacillus sphaericus* and *Ae. aegypti* strains

The *L. sphaericus* strains OT4b.25 and III(3)7 were isolated from beetle larvae and an oak forest soil, respectively [6]. The WHO reference strain 2362 was isolated from adult *Simulium damnosum* [26] and kindly donated by A. Delecluse from the Pasteur Institute in France. The larvae of the reference *Ae. aegypti* Rockefeller strain were kindly donated by the National Institute of Health (INS) in Bogotá, Colombia. To evaluate a field-collected strain, weekly samplings were conducted in twelve sectors of La Mesa, Cundinamarca (4°38'05.9"N, 74°27'45.4"W) where there is a temephos-resistant *Ae. aegypti* population [25].

### *Aedes aegypti* maintenance

Individuals donated by the INS were kept at 30 °C and 60–70% relative humidity under 12:12 light/dark photoperiod. The larvae were fed with pellet food Omega One Natural Protein Formula (OmegaSea, LLC, Painesville, USA) for cichlids, twice a week. Pupae were placed in a separate container with tap water until the adults emerged. The adults were transferred to 30 cm^3^ glass cages where they were continuously provided with a 10% sucrose solution to feed on. A week post-emergence, adults were deprived of sugar for 12 h and provided with lamb’s blood (©Microgen Ltda, Bogotá, Colombia) at 37 °C through an artificial feeder. The same conditions were provided to the field-collected strain.

### Bioassays of *L. sphaericus* against *Ae. aegypti* Rockefeller strain

Based on the LC_50_ found by Santana and coworkers (work submitted) for vegetative cells against *Ae. aegypti*, the three *L. sphaericus* strains were evaluated in a concentration of 10^7^ CFU/ml (Table 1). For the assays, the *L. sphaericus* strains were grown on nutrient agar (NA) at 30 °C. After 15 h, cells were recovered in 1 ml of water, and serial dilutions were then made to calculate the bacterial titer that was added to the bioassays. To assess the effect of *L. sphaericus* strains against each mosquito stage, the tests were conducted in 30 ml of chlorine-free water with 20 individuals from F1 generation and 300 μl of the bacterial suspension (10^9^ CFU/ml). Final bacterial concentrations are specified in Table 1. All the trials were made in triplicate and control without bacteria was included. For the eggs, a positive control with pellet food was carried out. The number of hatched eggs, live larvae, and adults that emerged from the pupae were recorded every 24 h until 48 h for larvae and pupae, and 72 h for eggs.

**Table 1:** *Lysinibacillus sphaericus* vegetative cells samples tested against *Aedes aegypti*

	Final bacterial concentration (± SD) CFU/ml		
*Ae. aegypti*	2362	III(3)7	OT4b.25
Rockefeller	1.43 × 10^7^ (± 9.06 × 10^5^)	1.52 × 10^7^ (± 6.43 × 10^6^)	2.04 × 10^7^ (± 5.17 × 10^6^)
Field-collected	1.73 × 10^7^ (± 6.04 × 10^6^)	1.79 × 10^7^ (± 5.77 × 10^6^)	2.49 × 10^7^ (± 11.26 × 10^6^)

*Abbreviations*: *SD* standard deviation

### Bioassays of a *L. sphaericus* consortium against *Ae. aegypti* Rockefeller and field-collected strains

A consortium made up by the three *L. sphaericus* strains was evaluated against eggs, the four larval stages, and pupae of the both *Ae. aegypti* strains. For each stage, the assays were conducted with the conditions mentioned previously, but the 300 μl of bacterial suspension was comprised of 100 μl of each *L. sphaericus* strain (10^9^ CFU/ml). Final bacterial concentrations tested for each *Ae. aegypti* strain are listed in Table 1. The assays were run in triplicate and control without bacteria was also included. The serial dilutions used to calculate the bacterial titer and the record times for individual survival in each stage were performed as described above.

### Preliminary assays of hemolytic and chitinase activity

Since hemolysins and chitinases genes were annotated in *L. sphaericus* genomes, *L. sphaericus* vegetative cells were assessed by their hemolytic and chitinase activity. Hemolysis was determined by the formation of halo on sheep blood agar (SBA). For that, *L. sphaericus* III(3)7, OT4b.25 and 2362 were cultured on SBA at 30 °C for 48 h. Chitinase activity was evaluated by the use of colloidal chitin as the only source of carbon [27, 28]. The three *L. sphaericus* strains were grown on minimal salt medium (MSM) comprised of KH_2_PO_4_ (0.5 g/l), Na_2_SO_4_ (2 g/l), KNO_3_ (2 g/l), CaCl_2_ (0.001 g/l), MgSO_4_ (1 g/l), FeSO_4_ (0.004 g/l), agar (15 g/l) and supplemented with 4% colloidal chitin. The plates were maintained at 30 °C for 72 h and then stained with 2% Congo red.

### Statistical analysis

To assess statistically significant differences between hatching, mortality, and adult emergence per treatment in the *Ae. aegypti* field-collected and Rockefeller strains, we performed ANOVA or Kruskal-Wallis non-parametric tests (when data were not normally distributed). A Tukey-Kramer test was performed to observe statistically significant differences between groups. A significance value of *P* < 0.05 was established for all tests. All the statistical analyses were realised in R [29].

## Results and discussion

### Activity of *L. sphaericus* on *Ae. aegypti* Rockefeller strain

**Fig. 1:**
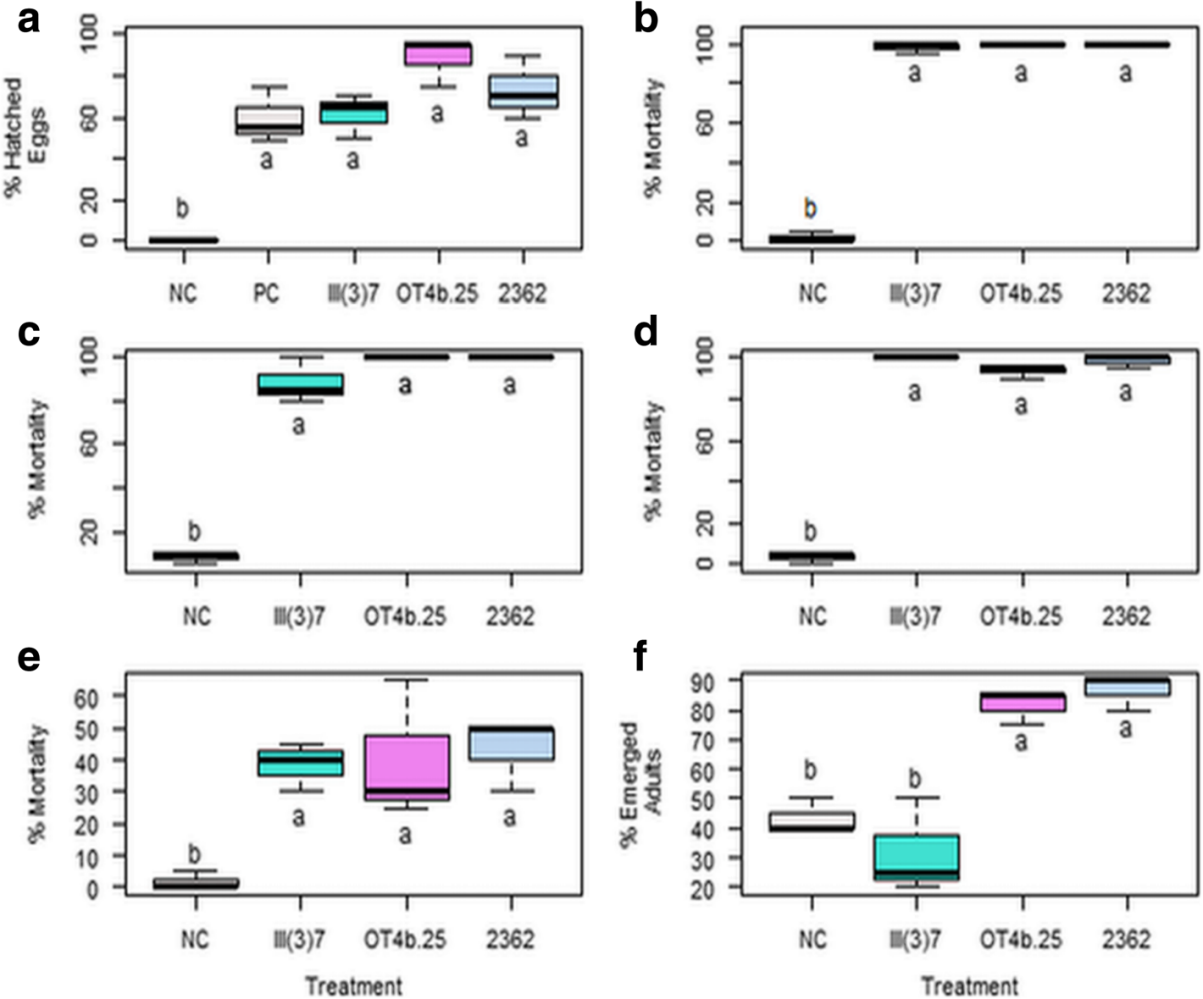
Effect of *L. sphaericus* III(3)7, OT4b.25, and 2362 vegetative cells (between 1.4 and 2 × 10^9^ CFU/ml) on the life-cycle stages of *Ae. aegypti* Rockefeller strain. **a** Percentage of eggs hatched at 72 h, the negative control had no food and the positive control was subject to pelleted food. Percentage of larvae mortality: **b**, first-instar; **c**, second-instar; **d**, third-instar; **e**, fourth-instar. **f** Percentage of emerged adults from pupae at 48 h. *Abbreviations*: NC, negative control; PC, positive control. Different letters under the boxes indicate statistical significance (*P* < 0.05)

The three *L. sphaericus* strains showed a similar percentage of hatched eggs compared to the positive control, and these groups were statistically different from the negative control, showing that treatment had no effects on egg hatching (Kruskal-Wallis: *χ* ^2^ *=* 10.71, *df = 4*, *P* = 0.02) (Fig. 1a). In the case of *L. sphaericus* OT4b.25, III(3)7, and 2362 strains, once the larvae hatched, all of them died within a period of no longer than 24 h. Although the eggs hatch when they are immersed in water with available oxygen, a food source is necessary [30, 31]. Therefore, in the first hours, the bacteria could be a potential food source but when the eggs hatch, the emerging larvae die because of the larvicidal activity of *L. sphaericus*. Ovicidal activity has been reported for plants and marine sponge extracts [32, 33], but the mode of action of this compounds on eggs has not been described. Since the hatched eggs percentage was high and very similar between the three *L. sphaericus* strains and the positive control, there is no ovicidal effect against *Ae. aegypti* eggs. It is well known that *L. sphaericus* crystal-spores are toxic only when these are ingested; however, the mode of action of the vegetative cells is still unknown. Despite this, the results suggest that the no-feeding stages such as eggs are not susceptible to *L. sphaericus*.

Regarding larval instars, the three *L. sphaericus* strains caused mortality, but the efficiency varied depending on the instar (Fig. 1b-e). For the first three larvae instars, the Tukey-Kramer test indicated that there were differences between the mortality caused by each bacterial strain and the number of dead larvae in the negative control (*P* < 0.001). A mortality of 100% occurred after 24 h in the first-instar; and for the second- and third-instar, this efficiency in mortality was reached after 48 h. In the fourth-instar, the mortality was 60% at 48 h, but the three strains (III(3)7, OT4b.25 and 2362) all had a negative effect against this stage (Tukey-Kramer: *P* = 0.3, *P* = 0.28, *P* = 0.018, respectively) (Fig. 1e).

For the pupae, we found statistically significant differences among groups (ANOVA: *F*_(3,8)_ = 25.21, *P* < 0.001). The percentage of emerged adults was similar for the OT4b.25 and 2362 strains, but it was different for the III(3)7 strain and the negative control (Fig. 1f). As the pupae are metabolically inactive, and fewer adults than expected emerged in the negative control, it is possible that the bacterial strains did not affect adult emergence. Reports of the effectivity of plants extracts and seaweeds on *Ae. aegypti* pupae suggests that these compounds contain toxic chemicals which cause mortality by contact or choking [34, 35]. As for the eggs, the results show the lack of activity of vegetative cells on pupae, suggesting that the samples tested do not act by contact on the non-feeding stages.

### Activity of the *L. sphaericus* consortium on the *Ae. aegypti* Rockefeller and field-collected strains

**Fig. 2:**
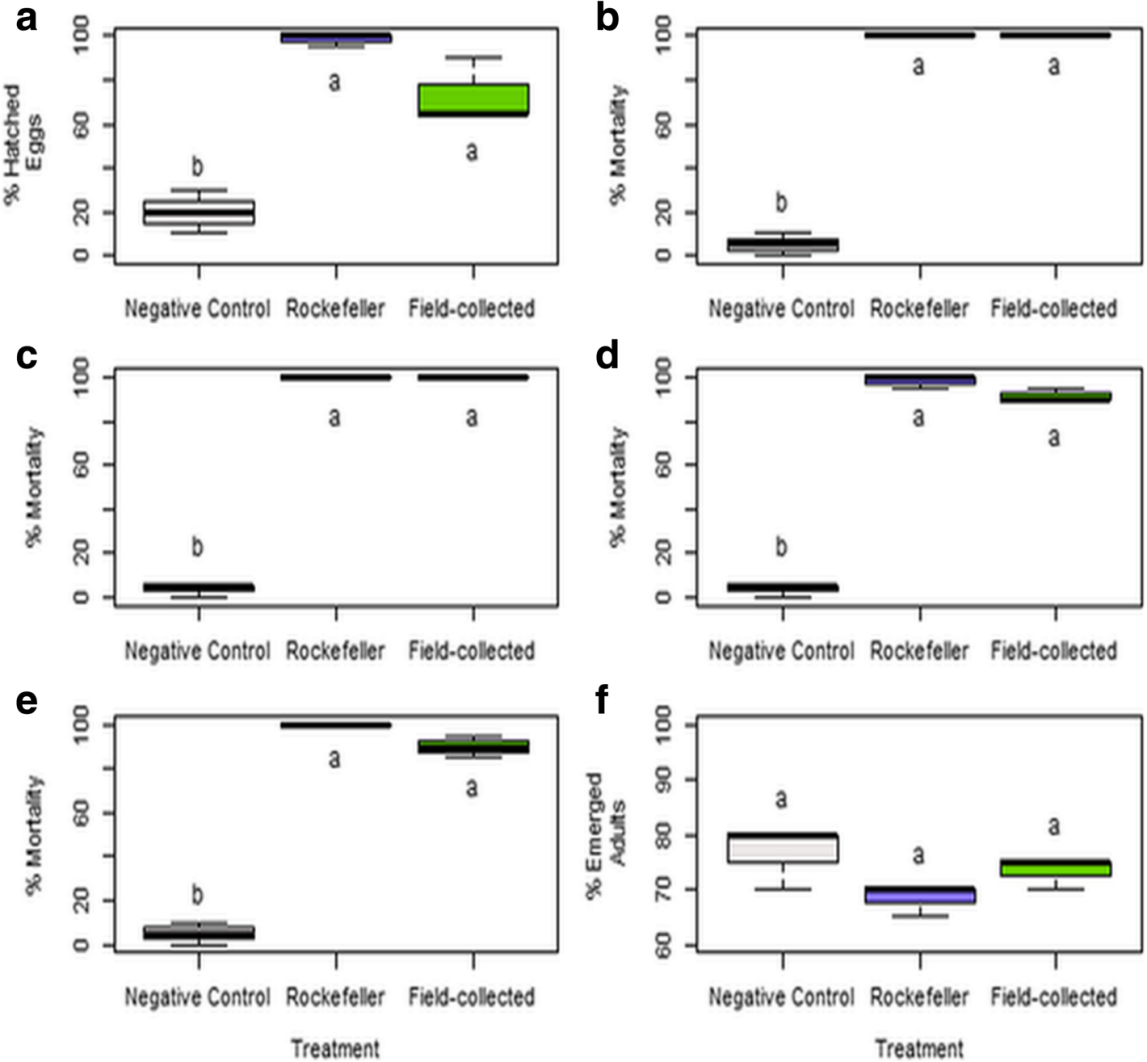
Effect of a consortium of *L. sphaericus* vegetative cells (between 1.7 and 2.5 × 10^9^ CFU/ml) on the life-cycle of the Rockefeller and temephos resistant *Ae. aegypti* (field-collected). **a** Percentage of eggs hatched after 72 h. Percentage of larvae mortality at 48 h: **b**, first-instar; **c**, second-instar; **d**, third-instar; **e**, fourth-instar. **f** Percentage of emerged adults from pupae at 48 h. Different letters under the boxes indicate statistical significance (*P* < 0.05)

Given that all the strains were very efficient against the reference Rockefeller strain, and that resistance mechanisms are increasingly presented by the mosquitoes, a bacterial consortium was evaluated against *Ae. aegypti* field-collected and Rockefeller strains. For the eggs, the bacteria consortium seems to act similarly as individual strains because no effects on eggs hatching were seen. We hypothesised that the consortium is a primary food source, but once the eggs hatch, the first larval stage died (Fig. 2a). The statistical difference between the negative control and the percentage of eggs of both *Ae. aegypti* strains (Tukey-Kramer: *P* = 0.001, *P* < 0.001), confirm the fact that if there is no food source, the eggs do not hatch.

For all the larvae instars, the consortium had a significant effect on larva development, causing 100% mortality at 48 h, for the two *Ae. aegypti* strains (Fig. 2b-e). There were significant differences between the mortality percentage in the control and the field-collected and Rockefeller strains (Kruskal-Wallis: *χ* ^2^ *=* 7.62, *df =* 2, *P* = 0.02). As for the pupae (Fig. 2f), there were no statistically significant differences between the control and the treatment with the bacterial consortium in both *Ae. aegypti* strains (ANOVA: *F*_(2,6)_ = 3.16, *P* = 0.115). According to these results, both the consortium and each bacterium strain on its own had no apparent effect on adult emergence, but a higher larvicidal activity as from 24 h.

The bacterial consortium had the same effect on the *Ae. aegypti* field-collected strain as on the Rockefeller strain and was more efficient than each strain evaluated separately. The better efficiency of *L. sphaericus* consortium was detected for fourth instar larvae in what there was 40% more mortality than that caused by individual strains. Since the *Ae. aegypti* field-collected strain is resistant to temephos and increasingly resistant populations of *Ae. aegypti* have appeared; the consortia of microorganisms are of great importance in the biological control of *Ae. aegypti* by overcoming the increase of resistance to commonly used larvicides. The organophosphate temephos is currently used due to its cost-effectiveness and the acceptance of the most vulnerable communities. However, the results herein presented suggest that *L. sphaericus* could be a better alternative when it comes to controlling *Ae. aegypti* populations; besides its effectivity, *L. sphaericus* is specific against culicids insects [36–38].

### Preliminary assays of hemolytic and chitinolytic activity

**Fig. 3:**
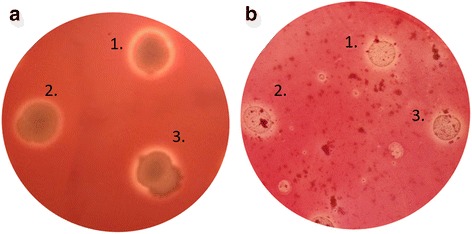
In vitro evidence of the hemolytic and chitinase activity of *L. sphaericus* strains. **a** Formation of halo on SBA at 48 h indicate hemolysis. **b** Halo around the growth areas indicate degradation of chitin on MSM supplemented with colloidal chitin. The numbers in the figure indicate *L. sphaericus* strains: *1*, III(3)7; *2*, 2362; *3*, OT4b.25

The three *L. sphaericus* strains exhibited hemolytic activity since 24 h, because of the formation of the transparent zone on SBA as is shown in Fig. 3a. Furthermore, due to Congo red staining is based on the differential union to chitin and there is a non-staining area around the grown of each strain, we could infer that all the strains used the colloidal chitin as carbon source Fig. 3b. In vitro assays to evaluate hemolytic and, chitinolytic activity has validated the genomic evidence regarding the presence of hemolysins and chitinases genes in some *L. sphaericus* strains. Although the mode of action of these toxic proteins is still unknown, the results herein presented reveals a possible key role of hemolysins and chitinases in the entomopathogenic activity of *L. sphaericus* vegetative cells. As the hemolysins of other entomopathogenic bacteria [39], the hemolysins annotated in *L. sphaericus* could be pore-forming enzymes and thus, these might be determinant virulence factors. Likewise, as other bacterial chitinases, those of *L. sphaericus* probably play a fundamental role in the hydrolysis of chitinous structures [40].

Other less toxic proteins produced by *L. sphaericus* are the Mtx toxins and Cry48-Cry49 binary protoxins. Mtx toxins are degraded into the vegetative cells [14], and Cry48-Cry49 are toxic for *C﻿.﻿* *quinquefasciatus* but not for *Ae. aegypti* [41]. Therefore, these proteins have not been used in *L. sphaericus*-based formulations, whose effectiveness relies on the high toxicity of *L. sphaericus* crystal-spores [42, 43]. Since *Ae. aeypti* larvae are refractory to Bin toxins and vegetative cells exhibit a diverse collection of toxic proteins, we suggest that *L. sphaericus* vegetative cells could be used to control *Ae. aegypti* populations. In point of fact, we hypothesised that the effect of the vegetative cells is due to a contribution of the toxic proteins harboured in *L. sphaericus* vegetative cells such as hemolysins, chitinases and S-layer protein. However, the way each protein contributes to the toxicity needs to be described in future studies.

The World Health Organization suggests that instar three is the proper stage to evaluate the larvicidal effect of a new substance [44]. Our results demonstrated the effectiveness of the three bacterial strains against any of the four larval stages, suggesting that *L. sphaericus* could potentially be used as a larvicidal agent. *Bacillus thuringiensis* serovar. *israelensis* is a bacterium widely used in the control of *Ae. aegypti* and other culicids [45]. Even the whole crystal of Bti is used in commercial formulations for mosquito control, resistance to some single Bti toxins have been appeared in recent years [46, 47]. Therefore, the compendium of toxic proteins of *L. sphaericus,* make this bacterium a feasible solution to the resistance of mosquitoes*.*

Although S-layer and Mtx toxins have shown entomopathogenic activity [16, 18], these do not have the properties of a biological controller. Meanwhile, the vegetative cells of *L. sphaericus* can persist and recycle in the environment. *L. sphaericus* persistence and recycling is possible because of its ability to grow under polluted environments and resist the UV light [48]. Likewise, bacteria go through a cycle where vegetative cells germinate from a dormant spore, which makes possible the infection by the vegetative cells even if there were spores in the larval environment. Other advantages of *L. sphaericus* include safety for humans and reductions of pesticide residues in the environment [48]. Likewise, some studies have developed an easy way of industrial production of *L. sphaericus* which is a concern when a formulation is accepted for a large treatment [49]. These features together with the results herein presented make *L. sphaericus* vegetative cells a suitable biological control alternative.

## Conclusions

In summary, the *Ae. aegypti* field-collected strain was susceptible to the vegetative cells of *L. sphaericus* which reveals the potential mosquitocidal activity of this bacterium. For a better understanding of the vegetative cells effect in *Ae. aegypti* larvae, future studies should be conducted to find out the role of chitinases, hemolysins and other toxic proteins genes annotated in *L. sphaericus* genomes. Given that the consortium was more efficient causing larvae mortality in *Ae. aegypti* resistant to temephos, different formulations should be assessed. The results indicate that the vegetative cells of *L. sphaericus* are a suitable alternative, which can be used to deal with insecticide resistance and to control vectors populations such as that of *Ae. aegypti*.

## Abbreviations

Bin: Binary toxin
BSA: Blood sheep agar
INS: National institute of health
MSM: Minimal salt medium
NA: Nutrient agar
